# Predictors of anemia in pregnant women residing in rural areas of the Oromiya region of Ethiopia

**DOI:** 10.1186/s40795-017-0166-y

**Published:** 2017-07-25

**Authors:** Krista Zillmer, Ashish Pokharel, Kathryn Spielman, Meghan Kershaw, Kidane Ayele, Yitbarek Kidane, Tefera Belachew, Robert F. Houser, Eileen Kennedy, Jeffrey K Griffiths, Shibani Ghosh

**Affiliations:** 1 Gerald J. and Dorothy R. Friedman School of Nutrition Science and Policy, Tufts University, 150 Harrison Ave, Boston, MA 02111 USA; 2 Jimma University, 378 Jimma, Ethiopia

**Keywords:** Pregnancy, Anemia, Ethiopia, Maternal, Nutrition

## Abstract

**Background:**

Anemia in pregnancy is associated with higher risk of low birth weight and both maternal and perinatal mortality. While previous studies in Ethiopia have examined factors associated with anemia, which factors are the most important determinants of anemia in this population remain unclear. The objective of this study was to examine the association between anemia status in pregnant women with different health, behavioral, and socioeconomic factors in Oromiya province of Ethiopia.

**Methods:**

This study used pregnancy enrollment data from a longitudinal birth cohort study conducted in Ethiopia. Survey data on maternal and household characteristics were collected at enrollment and maternal hemoglobin levels were measured. The analysis includes 4600 pregnant women. Logistic regression models were used to identify factors associated with maternal anemia in pregnancy.

**Results:**

Controlling for geographic location and religion, low maternal MUAC and previous pregnancies were associated with increased odds of anemia, with odds ratios of 1.30 (*p* < 0.001, CI 1.12–1.51), and 1.50 (*p* = 0.002, CI 1.16–1.95), respectively. For each additional point on the handwashing score scale, the odds of being anemic were reduced by 12% (*p* < 0.001, CI 0.82–0.94). Numerate women compared to non-numerate women had 30% lower odds (*p* < 0.001, CI 0.57–0.85).

**Conclusion:**

Controlling for woreda and religion, low maternal MUAC, and previous pregnancy increased odds of anemia while numeracy and better handwashing practices significantly reduced the odds of anemia in pregnancy. Further investigation is needed to determine the cause of anemia in pregnant women in Oromiya and to determine the effects of maternal anemia on birth outcomes.

## Background

Anemia is a major public health concern and prevalence remains high in many countries of South Asia and sub-Saharan Africa. Anemia during pregnancy is a critical issue as it is associated with higher risk of low birth weight and both maternal and perinatal mortality [1]. Anemia can be either nutritional (iron deficiency, folate and vitamin B12) or non-nutritional (parasitic infections, genetic diseases) in nature. Globally, it is estimated that iron deficiency is the cause of about half of anemia cases, though this will vary. Anemia due to folate and vitamin B12 deficiencies is also a significant public health burden, but prevalence rates vary by country and world region [2]. Pregnant women are considered anemic if they have a hemoglobin concentration less than 110 g/L [3].

Prevalence of anemia in pregnant women in Ethiopia was 22% in 2011 according to the Demographic and Health Survey (DHS) [4]. It varied by area of residence, with a higher prevalence of anemia in rural women (18%) than urban (11%) and geographical location, with prevalence ranging from 44% in the Somali region to 9% in Addis Ababa. It should be noted that the national and regional estimates in DHS 2011 are a significant improvement from 2006, where 62.7% of the pregnant women and 52.7% of non-pregnant women were reported to be anemic [5]. Other recent studies in Ethiopia have reported prevalence of anemia ranging from 16.6% in a facility-based study in Gondar, northwest Ethiopia to 56.8% in Gode town, Eastern Ethiopia [6, 7].

Prior studies in Ethiopia have reported significant associations between anemia in pregnancy and parasitic infections (e.g. schistosomiasis, hookworm infection), prior use of contraceptives, use of iron supplementation, birth spacing/intervals, parity and gravidity, educational attainment, age, body weight, trimester of pregnancy and wealth status. [6–18]. The relationship between anemia and dietary practices in Ethiopia has shown mixed results [12, 18, 19]. These findings indicate the need for an integrated approach to address the prevalence of anemia using both nutrition specific and nutrition sensitive approaches in targeting anemia in pregnancy. Multi-sectoral programs focus on improving maternal and infant and young child nutrition through programs and interventions that target improving food security, access to services and other related interventions.

Empowering New Generations to Improve Nutrition and Economic Opportunities (ENGINE), a five year USAID program implemented in four regions of Ethiopia (Oromiya, Tigray, Amhara and SNNPR) aimed to improve nutritional status of women and children through a multi-sectoral approach by coupling with the existing Agriculture Growth Program (AGP) to improve service delivery and utilization of services through training and strengthening of the health force, increasing awareness, and improving knowledge and practices around nutrition and health of mothers and primary caregivers.

A longitudinal birth cohort study implemented within the USAID ENGINE program between 2014 and 2016 aimed to investigate the effectiveness of the delivery of interventions targeting maternal and infant health and nutrition outcomes in three woredas (Woliso, Goma and Tiro Afeta) of the Oromiya region of Ethiopia. Approximately 4680 pregnant women were followed from recruitment in the second or third trimester through birth and their infant turning one year of age. Data were collected across different time points through the longitudinal study with data domains ranging from socio-economic status to household food security, maternal dietary recall, food taboos and consumption patterns, agricultural production, health status of the pregnant woman as well as measurements of anemia and malaria using HemoCue® and Rapid Diagnostic tests (RDT).

The aim of this study was to determine the prevalence of anemia in the pregnant women of the ENGINE cohort study and determine the factors associated with the increased risk of anemia. We examined nutrition specific (e.g. use of iron supplements, use of antenatal care, health and nutrition status, dietary diversity, household food security) and nutrition sensitive (e.g. crop and livestock production diversity, presence of malaria, water/hygiene and sanitation practices) factors that would potentially explain the odds of being anemic as a pregnant woman in a select population in Ethiopia.

## Methods

Data for this study were extracted from the longitudinal ENGINE Birth cohort study that was implemented from 2014 to 2016 in three woredas of Oromiya region in Ethiopia, two of which were part of the USAID ENGINE program while the third was not. The sample size for the Birth Cohort study was estimated at 4680 with 1560 women recruited in each woreda to allow for a comparison between woredas. Due to missing data for a few independent variables, 4600 women were included in our analysis sample. For the main study, the sample size calculation was based on the outcome of height for age Z-score. For this analysis, where the outcome is anemia, this sample allows us to detect an effect of 0.018 change in odds of anemia with 80% power at the 0.05 level of significance.

Pregnant women ages 14 to 50 years old were recruited from the three woredas from a total of 78 kebeles (*N* = 4680). Data were collected twice during pregnancy, at birth, and then every 3 months until the child reached 12 months of age. The data used in this study were obtained through surveys and assessments administered to the pregnant women during the time of recruitment. In addition, the present study used data collected from the survey administered to the household head at recruitment. Data was collected electronically through a tablet using Open Data Kit.

### Data calculations and variable definitions

Hemoglobin levels were measured with the HemoCue® system for mobile screening. Hemoglobin cutoff values were adjusted for altitude and trimester according to method described by Cohen and Hass [20]. The average kebele altitude was substituted for missing values for altitude. Women with hemoglobin levels below the adjusted cutoff point were classified as anemic. A binary variable for anemia status was used as the outcome variable in the analysis.

Marital status was categorized into 3 groups: married and monogamous, married and polygamous, and not married (single, cohabitating, separated, divorced, widowed). Most of the households recruited were Muslim or Orthodox (90%), thus religion was categorized as Muslim, Orthodox, or other. Women were classified as literate if they could read specific sentences in Oromifa and numerate if they correctly answered a simple math problem (i.e. "If you sell eggs for 30 Birr and chicks for 50 Birr, how many Birr do you have?"). A wealth index was constructed using polychoric principal component analysis to represent a composite measure of a household’s cumulative living conditions and then separated into quintiles. This method was the same as described by the Demographic and Health surveys for Ethiopia [21].

Mid-upper arm circumference (MUAC) was used as measure of nutritional status. The average of three MUAC measurements was calculated and then categorized as normal or low MUAC. A MUAC measurement less than 23 cm was classified as low MUAC [22]. The variable for antenatal care visits was coded as a binary variable for whether they have sought antenatal care (ANC). Alternatively, or in addition to clinic visits, some women may have been visited at home by a health extension worker. A binary variable was created for whether they received any home visits from health workers in the past year. Iron supplementation was coded as a binary variable, which is defined as the receipt or purchase of iron supplements during the current pregnancy. Likewise, a binary variable was created for receiving treatment for intestinal worms during the current pregnancy. Whether a woman has had previous pregnancies was coded as a binary variable (0 = first pregnancy, 1 = previous pregnancies). A handwashing score was computed using seven self-reported questions about the critical times for hand washing (when dirt is visible, after toilet use, after cleaning a child following defecation, before preparing food, before serving a meal, before eating, before feeding a child).

Minimum dietary diversity scores (MDD-W) were constructed from a 24-h qualitative recall as a proxy indicator for nutrient adequacy of the diet [23]. Foods were grouped into the following categories: all starchy staple foods, beans and peas, nuts and seeds, dairy, flesh foods, eggs, vitamin A-rich dark green leafy vegetables, other vitamin A-rich vegetables and fruits, other vegetables, and other fruits for a maximum score of 10. Household food insecurity access scale (HFIAS) score was constructed using the method described by Coates et al. [24].

Crop production diversity was calculated as a simple count of the crop groups produced annually by the household. Crops were grouped as cereals, roots and tubers, legumes, cash crops, vegetables, fruits, oil seeds, and spices for a maximum score of 8. Livestock production diversity was created as a count of products from livestock. The score was constructed from the following products: beef, milk, butter, cheese, cattle hides, cattle manure, yogurt, sheep meat, wool, sheep hides, sheep manure, goat meat, goat milk, goat hides, goat manure, eggs, bird manure, honey, wax, and propolis for a maximum score of 20.

Because recruitment occurred on a rolling basis, it was necessary to control for lean season. Months of adequate household food provisioning (MAHFP) were used to define the lean season. In our sample, the highest MAHFP scores, which signify the highest levels of food insecurity, occurred between June–September. A binary variable for lean season was created based on recruitment during those months. The variable for market access was defined as minutes to the nearest local or major market.

### Statistical analysis

Data were analyzed using Stata Corp 2013, StataSE 14 software. Descriptive statistics included generation of means and standard deviations along with bivariate analysis before variables were included in the model. A multivariate logistic regression analysis was conducted to ascertain the factors associated with being anemic in this population. The dependent variable was a binary variable of presence or absence of anemia (prevalence) while the independent variables included in the model were lean season, presence of low MUAC, previous pregnancy, trimester, number of antenatal visits to the clinic (by the pregnant woman), number of health worker home visits, use of iron supplementation, use of deworming, handwashing score, age, market access, HFIAS, wealth quintile, minimum dietary diversity score, crop and livestock production diversity, woman’s literacy and numeracy. The model was adjusted for clustering at the kebele level, woreda (this also controlled for presence or absence of ENGINE as an intervention) and religion and includes robust standard errors using the vce command in Stata. A *p*-value of less than 0.05 was considered as a statistically significant result.

After the preliminary model, multiple iterations were tested including the removal of insignificant variables and addition of interaction terms. However, neither the inclusion of interaction terms nor the removal of insignificant variables improved the model. Furthermore, the presence of interaction terms worsened the model as determined by Akaike’s and Schwarz’s Bayesian information criteria (AIC/BIC) using the estat ic command in Stata for assessing the model fit. Adjusted odds ratios and 95% confidence intervals are reported.

## Results

### Anemia prevalence

The prevalence of anemia, adjusted for altitude and trimester, was 24.09% for the entire sample (Table 1). Goma had the lowest prevalence at 19.57%, followed by Woliso at 20.29% and Tiro Afeta with a prevalence of 32.52%. The mean hemoglobin was 12.5 g/dL (±1.35). A total of 4600 women were included in the final analysis.

**Table 1:** Maternal Characteristics

	Mean (sd)/Number of women reporting (%), overall and by woreda				
	Goma (*n* = 1548)	Tiro Afeta (*n* = 1519)	Woliso (*n* = 1533)	Total (*n* = 4600)	*p*-value
Anemia	303 (19.57%)	494 (44.58%)	311 (28.07%)	1108 (24.09%)	0.000
Hemoglobin	12.56 (1.30)	12.15 (1.47)	12.66 (1.20)	12.46 (1.35)	0.000
Age	26.17 (5.44)	26.51 (5.82)	26.56 (5.46)	26.41 (5.58)	0.104
Religion					
Muslim	1384 (89.41%)	1478 (97.30%)	234 (15.26%)	3096 (67.30%)	0.000
Orthodox	147 (9.50%)	26 (1.71%)	867 (56.56%)	1040 (22.61%)	0.000
Other	17 (1.10%)	15 (0.99%)	432 (28.18%)	464 (10.09%)	0.000
Literate	623 (40.25%)	241 (15.87%)	489 (31.90%)	1353 (29.41%)	0.000
Numerate	1417 (91.54%)	1219 (80.25%)	1435 (93.61%)	4071 (88.50%)	0.000
MUAC	23.97 (2.37)	22.98 (1.99)	23.53 (1.99)	23.50 (2.16)	0.000
Low MUAC	523 (33.79%)	773 (50.89%)	574 (37.44%)	1870 (40.65%)	0.000
Number of previous pregnancies	2.90 (2.35)	3.79 (2.79)	2.95 (2.32)	3.21 (2.53)	0.000
Previously Pregnant	294 (18.00%)	193 (12.71%)	285 (18.59%)	772 (16.78%)	0.000
Sought ANC this pregnancy	860 (55.56%)	640 (42.13%)	827 (53.95%)	2327 (50.59%)	0.000
Has had a home visit from an HEW	142 (9.17%)	259 (17.05%)	50 (3.26%)	451 (9.80%)	0.000
Received iron supplements	252 (16.28%)	316 (20.80%)	208 (13.57%)	776 (16.87%)	0.000
Received drug for intestinal worms	15 (0.97%)	25 (1.65%)	16 (1.04%)	56 (1.22%)	0.174
Handwashing score	5.04 (1.19)	4.10 (0.99)	3.63 (1.20)	4.26 (1.27)	0.000
Dietary diversity score	2.47 (0.79)	2.48 (0.69)	2.16 (0.73)	2.37 (0.75)	0.000

**Table 2:** Household characteristics, overall and by woreda

	Mean (sd)/Number of households reporting (%)				
	Goma (*n* = 1548)	Tiro Afeta (*n* = 1519)	Woliso (*n* = 1533)	Total (*n* = 4600)	*p*-value
Wealth Quintile					
First	77 (4.97%)	279 (18.37%)	594 (38.75%)	950 (20.65%)	
Second	74 (4.78%)	488 (32.13%)	347 (22.64%)	909 (19.76%)	
Third	229 (14.79%)	435 (28.64%)	239 (15.59%)	903 (19.63%)	
Fourth	505 (32.62%)	252 (16.59%)	218 (14.22%)	975 (21.20%)	
Fifth	663 (42.83%)	65 (4.28%)	135 (8.81%)	863 (18.75%)	
HFIAS^a^	4.38 (4.50)	5.09 (5.59)	4.39 (5.21)	4.62 (5.12)	0.000
HFIA Category					
Food Secure	10 (0.88%)	4 (0.40%)	2 (0.22%)	16 (0.53%)	
Mildly Food Insecure	262 (23.19%)	193 (19.17%)	134 (14.79%)	589 (19.36%)	
Moderately Food Insecure	690 (61.06%)	548 (54.42%)	578 (63.80%)	1816 (59.68%)	
Severely Food Insecure	168 (14.87%)	262 (26.02%)	192 (21.19%)	622 (20.44%)	
Lean Season	697 (45.03%)	689 (45.36%)	556 (36.27%)	1942 (42.22%)	0.000
Time to Market (min)	38.41 (33.75)	45.49 (29.99)	37.67 (34.78)	40.50 (33.09)	0.000
Crop Production Diversity Score	2.64 (1.38)	2.92 (1.86)	3.29 (1.46)	2.95 (1.60)	0.000
Livestock Production Diversity Score	1.25 (1.94)	1.34 (1.96)	2.67 (2.33)	1.75 (2.18)	0.000

^a^Household food insecurity access scale (HFIAS)

### Demographic and health characteristics

Tables 1 and 2 presents the descriptive statistics of the key factors that could affect anemia status in pregnant women. The majority of the women were between the ages of 20–29 (57%) and most of the women were in a monogamous marriage (96%). The women were primarily Muslim (67%) and had low literacy (30%) but had higher numeracy (88%). The majority of women from Goma and Tiro Afeta were Muslim (89%, 97% respectively), but women in Woliso were mostly Christian, including Orthodox, Catholic, and Protestant (85%). The was also large difference in literacy between woredas. Tiro Afeta had much lower literacy rates (16%), than Goma (40%) and Woliso (32%).

A large proportion of women had low MUAC scores (41%) and had not yet attended any antenatal care appointments during pregnancy (49%). Fewer women in Tiro Afeta had attended any ANC appointments (42%), than in Goma (56%) and Woliso (51%), but they did receive more home visits from health workers (17%, 9%, and 10%, respectively). Moreover, a significant proportion of the women did not buy or receive iron supplements during the current pregnancy (83%). The mean score for HFIAS was 4.61 on a scale of 0–27. These characteristics varied by woreda, and Goma typically fared better than the Tiro Afeta and Woliso woredas.

### Dietary diversity

We found low variability in diet diversity scores in our sample. The majority of women (85%) consumed either 2 or 3 food groups. Staple cereals and roots/tubers were consumed by 99.6% of the sample with 77% of the women reporting consumption of teff, followed by legumes at 63.1%, and dark green leafy vegetables (e.g. kale) at 33.5%. On the scale of 0–10, no women consumed more than 6 food groups. Consumption of animal source foods was rare, with only 11% of women consuming dairy, eggs, or meat.

### Logistic regression

Adjusting for the clustering at the kebele level and controlling for woreda and religion, we find factors that are positively associated (thus protective) with anemia include hand washing and numeracy (Table 3). All else equal, for each additional point on the handwashing score scale, the odds of anemia were reduced by 12% (*p* < 0.001). Women who were numerate were 30% less likely to be anemic (*p* < 0.001), holding all other variables constant. Interestingly, numeracy but not literacy was associated with decreased odds of anemia.

**Table 3:** Odds ratios from multivariate logistic regression predicting anemia

	Adjusted odds ratio	95% confidence interval	*p*-value
Household Characteristics			
Lean season	0.80**	0.67, 0.94	0.006
Goma	0.60**	0.46, 0.78	0.000
Woliso	0.74*	0.54, 0.997	0.048
Wealth quintile	1.03	0.97, 1.10	0.219
HFIAS^a^	1.02**	1.01, 1.04	0.007
Time to Market (min)	1.00	1.00, 1.00	0.262
Crop Production Diversity Score	1.04	0.99, 1.10	0.131
Livestock Production Diversity Score	0.98	0.94, 1.03	0.441
Maternal Characteristics			
Age (years)	1.00	0.99, 1.02	0.561
Literate	1.06	0.89, 1.27	0.505
Numerate	0.70**	0.57, 0.85	0.000
Trimester	0.90	0.76, 1.07	0.229
Low MUAC	1.30**	1.12, 1.51	0.000
Previously Pregnant (0 = First, 1 = Has been previously pregnant)	1.50**	1.16, 1.95	0.002
Has sought ANC this pregnancy	1.17	0.99, 1.39	0.072
Has had a home visit from an HEW	1.06	0.85, 1.32	0.633
Use of iron supplements	0.81	0.65, 1.01	0.063
Received drug for intestinal worms	1.14	0.59, 2.20	0.702
Handwashing score	0.88**	0.82, 0.94	0.000
Dietary diversity score	1.05	0.94, 1.17	0.355
Muslim	1.66**	1.22, 2.26	0.001
Other religion	1.38*	1.03, 1.85	0.029

Notes: *Statistical significance at *p* < 0.05, **﻿statistical significance at *p* < 0.01﻿; ^a^Household food insecurity access scale; Reference category for religion was Orthodox and the reference category for woreda was Tiro Afeta

Factors that increased the odds of anemia were low MUAC score and previous pregnancy (Table 3). All else equal, women with low MUAC were 1.30 times more likely to be anemic (*p* < 0.001). Compared to women who were pregnant with their first child, women who have had any prior pregnancies have 1.50 times the odds of being anemic (*p* = 0.002). While HFIAS was statistically significant in the model, the result is not practically meaningful (OR 1.02, *p* = 0.007).

## Discussion

The results of our study show that low MUAC (cutoff of <23 cm), previous pregnancy, numeracy (function of education), and handwashing were associated with maternal anemia. While prior studies across Ethiopia have shown significant associations between anemia and factors such as age, wealth status, dietary diversity, pregnancy trimester, number of antenatal care visits, and iron supplementation, our study found no significant effect of these variables on the odds of anemia [7, 17, 18].

Low MUAC was strongly correlated with anemia. Both nutrient deprivation and occurrence and re-occurrence of infections contribute to poor nutritional status. In our study sample, 28% of women with a low MUAC value were classified as anemic, but the odds of being anemic increased significantly when the mothers had low MUAC when compared to those who did not. The effect of MUAC on anemia status is consistent with findings of another recent study in Ethiopia, which demonstrated that a MUAC measurement above 23 cm reduced odds of anemia by 59% [7].

Like other studies, we did not find a relationship between anemia in pregnant Ethiopian women and dietary diversity, although dietary diversity has been shown to be associated with improved micronutrient adequacy [12, 18, 19, 25]. However, dietary diversity was low for nearly all women in our sample. About 80% of pregnant women in our sample reported consumption of teff in the past week, which is a staple grain unique to Ethiopia and naturally high in iron. Given the high levels of iron in teff (12.5 mg/100 g), we speculate that iron intake in this population might have been sufficient [26]. An in-depth diet assessment analysis is underway to determine the iron availability in their diets.

This study did find a significant association of likelihood of being anemic and those who have had prior pregnancies. These findings are supported by previous analyses in Ethiopia [6, 7, 10, 15]. Pregnancy increases the requirements for iron in the body and often multiple pregnancies deplete the iron stores in the women’s body, especially if they are closely spaced. Furthermore, higher scores on self-reported hand-washing habits significantly reduced the odds of anemia. The mechanism by which this works is likely by the prevention of infections such as those caused by intestinal parasites [27, 28]. Numeracy was also associated with reduced odds of anemia, the results of which are supported by previous studies that show an association between education and anemia [18, 29]. Interestingly, numeracy but not literacy was associated with anemia status.

Previous reports have demonstrated poor utilization of antenatal care and low coverage and compliance of iron/folic acid supplementation in Ethiopia [30, 31]. Increased utilization of antenatal care services and home visits by health extension workers present opportunities to improve coverage and compliance of iron supplementation and perhaps more importantly, to increase coverage of deworming treatment. In our study, anemia was not significant associated with iron supplementation and deworming treatment. Based on respondent reports, coverage of iron supplementation was low (16.9%) and coverage of deworming was even lower (1.22%). It should also be noted that subjects were tested for malaria, but the prevalence of malaria was very low (0.02%). It was important to examine the prevalence of malaria in this population as the study was conducted in a known malaria-endemic area of Ethiopia [32].

Anemia in pregnancy is a significant public health concern and coverage of iron supplementation remained low from the time of enrollment (17%) through the end of the pregnancy (45%). In addition to a low percentage of women who reported buying or receiving iron supplements, the average number of tablets taken by those women during pregnancy was 41, which is far below the recommended quantity of 180 tablets.

Prior studies have shown that anemia in Ethiopia is more often linked to infections such as malaria and hookworm and helminths [6, 14]. A study in southwest Ethiopia reported that 41% of the pregnant women in their sample had soil transmitted helminth infection [14]. While we find no relationship between anemia status and use of deworming medication, this study cannot account for the relationship of anemia and presence of helminths and hookworm infections, a common cause of anemia in Ethiopia, due to the lack of data. While this could be considered as a caveat in this study, we do include variables in the analysis (e.g. handwashing score) that could serve as a proxy for the presence of infections. Our study did not collect fecal samples to assess presence of helminths and hookworm, thus our conclusions are speculative.

## Conclusion

Factors associated with anemia in this population were household food insecurity, low maternal MUAC, previous pregnancy, numeracy, and handwashing practices. Anemia due to iron deficiency and other nutritional causes were likely to be low with non-nutritional causes being the more prevalent. Preliminary analysis of data from the time of birth and beyond showed that the prevalence of anemia among women in our sample increased from the time of recruitment during pregnancy (24%) to the time of birth (41%) and remained high one year after giving birth (23%). Future analysis will need to examine the effect of being anemic in pregnancy on birth outcomes and the interactions of being anemic in pregnancy with anemia at time of birth and beyond.
